# Trastuzumab does not bind rat or mouse ErbB2/neu: implications for selection of non-clinical safety models for trastuzumab-based therapeutics

**DOI:** 10.1007/s10549-021-06427-w

**Published:** 2021-10-27

**Authors:** Gail Lewis Phillips, Jun Guo, James R. Kiefer, William Proctor, Daniela Bumbaca Yadav, Noel Dybdal, Ben-Quan Shen

**Affiliations:** 1 Department of Discovery Oncology, Genentech, Inc., 1 DNA Way, South San Francisco, CA 94080 USA; 2 Department of Structural Biology, Genentech, Inc., South San Francisco, CA USA; 3 Department of Safety Assessment, Genentech, Inc., South San Francisco, CA USA; 4 Department of Preclinical and Translational Pharmacokinetics, Genentech, Inc., South San Francisco, CA USA; 5 Department of Pharmacokinetics, Pharmacodynamics, and Drug Metabolism, Merck & Co, Inc., South San Francisco, CA USA

**Keywords:** Trastuzumab, Trastuzumab emtansine, T-DM1, Binding, Human, Rodent

## Abstract

**Purpose:**

Assessment of non-clinical safety signals relies on understanding species selectivity of antibodies. This is particularly important with antibody–drug conjugates, where it is key to determine target-dependent versus target-independent toxicity. Although it appears to be widely accepted that trastuzumab does not bind mouse or rat HER2/ErbB2/neu, numerous investigators continue to use mouse models to investigate safety signals of trastuzumab and trastuzumab emtansine (T-DM1). We, therefore, conducted a broad array of both binding and biologic studies to demonstrate selectivity of trastuzumab for human HER2 versus mouse/rat neu.

**Methods:**

Binding of anti-neu and anti-HER2 antibodies was assessed by ELISA, FACS, IHC, Scatchard, and immunoblot methods in human, rat, and mouse cell lines. In human hepatocytes, T-DM1 uptake and catabolism were measured by LC-MS/MS; cell viability changes were determined using CellTiter-Glo.

**Results:**

Our data demonstrate, using different binding methods, lack of trastuzumab binding to rat or mouse neu. Structural studies show important amino acid differences in the trastuzumab-HER2 binding interface between mouse/rat and human HER2 ECD. Substitution of these rodent amino acid residues into human HER2 abolish binding of trastuzumab. Cell viability changes, uptake, and catabolism of T-DM1 versus a DM1 non-targeted control ADC were comparable, indicating target-independent effects of the DM1-containing ADCs. Moreover, trastuzumab binding to human or mouse hepatocytes was not detected.

**Conclusions:**

These data, in total, demonstrate that trastuzumab, and by extension T-DM1, do not bind rat or mouse neu, underscoring the importance of species selection for safety studies investigating trastuzumab or trastuzumab-based therapeutics.

**Supplementary Information:**

The online version contains supplementary material available at 10.1007/s10549-021-06427-w.

## Introduction

Overexpression of the HER2/ErbB2 receptor tyrosine kinase occurs in a subset of breast cancer, as well as other solid tumor types [1] and is correlated with poor clinical outcome [2]. Approved HER2-targeted therapies include antibodies (trastuzumab [3, 4], pertuzumab [5, 6]), antibody–drug conjugates (ADCs), such as trastuzumab emtansine (T-DM1) [7, 8] and trastuzumab deruxtecan [9], as well as small molecule kinase inhibitors [10, 11]. Safety assessment is a key part of preclinical and clinical drug development, with species selection for non-clinical studies key to interpretation of findings. For kinase inhibitors, use of non-primate models is acceptable due to similar homology of different kinases among species [12]. However, most monoclonal/humanized antibodies raised in rodent species against human antigens do not cross-react with the corresponding rodent antigen. This necessitates specific binding studies across different species (e.g., mouse, rat, dog, non-human primates) to determine appropriate species for assessment of target-dependent versus target-independent effects. Safety studies for antibody therapeutics are, in general, performed in non-human primates for target-dependent safety signals, and in rodents to assess antigen-independent safety.

The original paper describing generation of HER2 monoclonal antibodies (MAbs) [13] that resulted in humanization of MAbs 4D5 and 2C4 to produce trastuzumab and pertuzumab, respectively, stated, but did not show data, that these MAbs did not cross-react with rodent ErbB2/neu. Subsequent studies investigating the pertuzumab binding interface with HER2 demonstrated selectivity of pertuzumab for human HER2 versus neu. Of the 5 amino acids in HER2 extracellular domain (ECD) sub-domain II where pertuzumab binds that differ between human and rat ErbB2, only 2 make contact with pertuzumab. Substitution of the 2 rat neu residues into human HER2 resulted in complete loss of pertuzumab binding [14]. No such studies have been performed for trastuzumab-HER2 binding. Given the lack of robust structural and binding data, numerous investigators have used mice to study toxicities of trastuzumab and T-DM1.

Cardiac toxicity is the most serious adverse event reported in patients treated with trastuzumab [3]. Despite a number of proposed mechanisms, most studies have been carried out in mice [15–19] and must be interpreted with caution as trastuzumab does not bind mouse ErbB2/neu. Models for directly investigating trastuzumab cardiotoxicity are extremely limited, and are primarily human cardiomyocyte cultures [17, 18, 20]. One robust model for examining the function of erbB2 in adult cardiac tissue is the erbB2 conditional gene knock-out mouse with cardiac-restricted inactivation of erbB2 [21]. However, this model cannot be used to assess direct effects of trastuzumab.

Thrombocytopenia and transaminitis [7] are the most frequent adverse events for T-DM1. T-DM1-induced thrombocytopenia is not HER2-mediated, but is due to either trastuzumab binding Fcγ receptors or via micropinocytosis [22–24], a non-target-dependent mechanism. The mechanisms by which T-DM1 induces hepatotoxicity have not been adequately described, again, due largely to lack of appropriate preclinical models. Reports of T-DM1-induced TNF-α release, inflammation and necrosis in mouse liver mediated by HER2 [25] are inconsistent with the inability of trastuzumab to bind mouse or rat neu.

To address these issues, we assessed binding of trastuzumab or anti-neu antibodies to human HER2 and rodent neu utilizing different assays. Mutational studies were performed in which amino acids in the trastuzumab/HER2 binding interface in human HER2 were mutated to the corresponding residues in neu. Additionally, studies were performed in human hepatocytes to assess T-DM1 induced cell viability changes, uptake and catabolism. Results from binding, biochemical, structural and biologic assays demonstrate no specific binding or biologic effects of T-DM1 in cells expressing rodent neu, as well as no target-dependent uptake or catabolism in human hepatocytes. These data confirm the observations from Fendly et al. [13] and firmly establish the importance of using non-rodent species to explore mechanisms of toxicity of trastuzumab-based therapies.

## Materials and methods

### Supplemental materials and methods

Cell lines, reagents, FACS and IHC.

#### Cell lines and reagents

Sources of all cell lines, cell culture media, experimental reagents and antibodies can be found in the Supplemental section.

#### Fluorescence-activated cell sorting (FACS)

Binding of anti-HER2 and anti-neu antibodies to HER2 or neu on different human, rat and mouse cell lines was performed by FACS analysis.

#### Immunohistochemistry (IHC)

Immunohistochemical detection of HER2 and neu expression, utilizing anti-HER2 and anti-neu, antibodies, was performed on 3T3 fibroblast cells transfected to express high levels of human HER2 or rat neu.

### Generation of anti-neu monoclonal antibodies

Balb/c mice were injected weekly for 4 weeks with 50 μg gD-tagged rat c-neu in Ribi adjuvant in the rear footpad. On day 26, blood was collected for development and isolation of hybridoma monoclonal antibodies as previously described [13] Antibody reactivity was evaluated by ELISA (enzyme-linked immunosorbent assay) as follows. NUNC Maxisorb plates were coated with 1 μg/mL gD-neu, human HER2 ECD, gD-human HER3 ECD (extracellular domain) or gD-human HER4 ECD at 4 °C overnight. Plates were washed 3 times with assay buffer (0.5% BSA, 0.05% Tween 20 and 0.01% thimerosal in PBS), blocked with assay buffer, and biotinylated MAbs added for 1 h at room temperature. Samples included biotinylated anti-neu MAbs (all at 1:1000), as well as biotinylated MAb 7.16.4 for a positive control; biotinylated anti-HER2 4D5, anti-HER3 1511 and anti-HER4 1440. Signal was detected with streptavidin-HRP (Behringer Mannheim) using 405 nm reference/490 nm absorbance.

### ^125^I-labeled antibody binding studies

Detailed experimental methods are in the Supplemental Section. For Scatchard binding competition assays, cells were incubated with serial dilutions of unlabeled trastuzumab in the presence of a fixed concentration of ^125^I-trastuzumab, washed, and radioactivity measured by gamma counting.

Specificity of trastuzumab and MAb 2009 binding to human HER2 or rodent neu was determined using SK-BR-3 and DHFR-G8 cells incubated with either ^125^I-trastuzumab or ^125^I-MAb 2009, in the presence or absence of unlabeled trastuzumab and MAb 2009.

### HER2 mutant cell lines

CHO-K1 cells were transfected with wildtype or mutant (P579S, P593S, P594S, F595S) HER2 constructs using Lipofectamine 3000 (Invitrogen) according to manufacturer’s instruction. Stable transfectants were selected under 5 μg/mL puromycin for 7 days and were then sorted by FACS for high HER2-expressing cells.

### Immunoblot analysis

Immune precipitation and western blot analyses were performed two ways: using the De Lorenzo method [26] and our established method (described below). All cells were detached using Cell Dissociation Solution. For the De Lorenzo method, lysates were prepared by resuspending 7.5 million cells in 0.5 mL of lysis buffer (10 mM Tris–HCl, pH 7.4; 150 mM NaCl; and 0.5% NP-40 containing Complete, EDTA (ethylenediaminetetraacetic acid)-free protease inhibitor cocktail). For our method, lysates were prepared with 3 million cells/mL in 50 mM HEPES, pH 7.2, in RPMI; 1.0% v/v Triton X-100, 1.0% w/v CHAPS (cholamidopropyl-dimethylammonio-propanesulfonate) and Complete, EDTA-free protease inhibitor cocktail. For immune precipitation (IP), equal amounts of cell equivalents were used (i.e., 0.1 mL cells for De Lorenzo method versus 0.5 mL for ours). IP antibodies used were trastuzumab, MAb 2009 or normal human Ig at a concentration of 2 μg each. Samples were run through Protein-A/G Ultralink resin, separated using SDS-PAGE, and HER2/neu detected with antibody D8F12 XP which recognizes both human HER2 and rodent c-neu.

### Cell viability assays

Cryopreserved primary human hepatocytes were thawed in thawing medium at 37 °C, pelleted, and resuspended in plating medium. Viable hepatocytes were counted by trypan blue dye exclusion, seeded into black-walled, collagen-coated 96-well plates (Corning Life Sciences) at 50,000 cells/well in plating medium supplemented with 1% antibiotics and 5% FBS and incubated overnight to allow cells to adhere. Cells were then treated for 72 h with T-DM1, anti-gD-DM1 (anti-Herpes Simplex Virus glycoprotein D, non-targeted ADC), or trastuzumab diluted in 100 μL of serum-free medium. Cell viability was determined using CellTiter-Glo™ Assay following the manufacturers protocols. Luminescence was read on an EnVision Multi-plate Reader (PerkinElmer) and data were reported as percent of vehicle control wells, with 3 replicates per condition. Statistically significant differences between treatment groups were determined by 2-way ANOVA with multiple comparisons between mean values for each treatment group within each hepatocyte lot tested.

### Uptake and catabolism studies

Human primary hepatocytes cultured in 6-well plates were treated with 200 μg/mL of unlabeled T-DM1 or anti-gD-DM1 for 0–96 h. At time 0, 6, 24, 48, 96 h post treatment, the medium was removed, cells washed three times with cold PBS and collected in cell lysis buffer containing protease inhibitors, homogenized, and then extracted three times with the ethyl acetate/methanol extraction method as previously reported [27]. The ethyl acetate/methanol extracts were pooled after each extraction and collected in a 96-deep-well plate. Soluble fractions were evaporated to dryness in a TurboVap. The dried extracts were reconstituted in 0.12 mL of 80/20 aqueous ACN/water + 25 mM catechol. Samples were analyzed by LC-MS/MS (Liquid Chromatography with tandem Mass Spectrometry) for catabolites DM1, MCC-DM1, and Lys-MCC-DM1. The LC-MS/MS assay for MCC-DM1 had a lower limit of quantification (LLOQ) of 1.95 nM (1.90 ng/mL), for Lys-MCC-DM1, the LLOQ value was 1.95 nM (2.15 ng/mL), and for DM1-NEM, the LLOQ was 0.24 nM (0.18 ng/mL).

Catabolites concentrations (nM) were further converted to the total amount of catabolites, pM per well per 10^6^ cells, using the following formula:

$${\text{Total}}\,{\text{catabolite}}\,\left( {{\text{pM}}} \right)\,{\text{per}}\,{\text{well}}\,\left( {10^{6} } \right) = \frac{{{\text{Concentration}}\,{\text{measured}}\,\left( {{\text{nM}}} \right) \times 1000}}{{1000\,{\text{mL}}}} \times {\text{Total}}\,{\text{sample}}\,{\text{volume}}$$

## Results

### Generation and characterization of anti-c-neu monoclonal antibodies

**Fig. 1:**
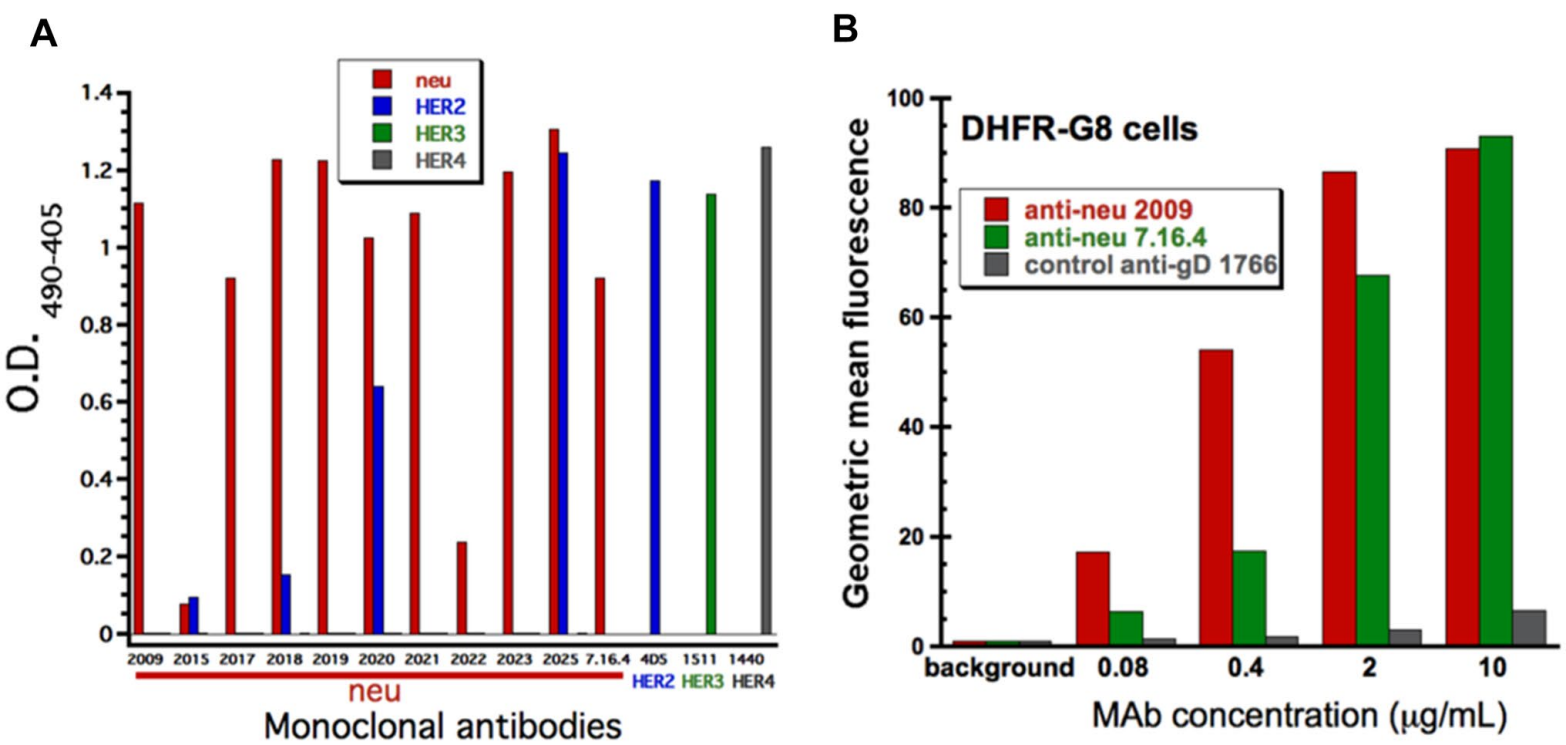
Anti-neu antibody binding assessed by ELISA and FACS. **A** Binding of neu antibodies to neu, HER2, HER3, or HER4 by ELISA. **B** Comparison of MAb 2009 binding versus MAb 7.16.4 on DHFR-G8 cells by FACS

Given the limited availability of anti-neu antibodies, we developed hybridoma-derived anti-c-neu monoclonal antibodies (MAbs) from mice immunized with gD (herpes simplex virus glycoprotein D)-tagged c-neu. Binding of 10 biotinylated c-neu MAbs to gD-neu-, HER2 ECD-, gD-HER3 ECD- or gD-HER4 ECD-coated plates was characterized using ELISA. Eight out of 10 c-neu MAbs, demonstrated comparable or better binding to neu compared to the positive control 7.16.4 (MAbs 2009, 2017, 2019, 2020, 2021, 2023 and 2025; Fig. 1A). MAbs 2020 and 2025 also demonstrated binding to human HER2, as compared to the positive HER2 control, muMAb 4D5. MAb 2015 showed weak binding to c-neu and HER2; low neu-binding was also observed with MAb 2018. No binding to HER3 or HER4 by any neu MAb was detected. For MAbs 7.16.4, 4D5, 1511 and 1440, binding was confirmed on their cognate receptors (c-neu, HER2, HER3 and HER4, respectively). Out of the 4 MAbs that demonstrated optimal neu-binding profiles (2009, 2019, 2021, 2023, MAb 2009 was selected for further characterization. DHFR-G8 cells are NIH/3T3 cells engineered to express high levels of c-neu [28]. Flow cytometry was utilized to compare binding of MAb 2009 versus 7.16.4 in a live-cell FACS assay. Concentration-dependent increases in binding to DHFR-G8 cells was demonstrated with both MAbs, with greater binding sensitivity for MAb 2009 (Fig. 1B). As mice were immunized with gD-tagged neu, it was important to verify no binding by anti-gD MAb 1766. These data confirm comparable binding properties of our anti-neu MAbs compared to the well-characterized MAb 7.16.4 [29, 30].

### Immunohistochemical analysis of anti-HER2 muMAb 4D5 and anti-neu MAb 7.16.4 binding to HER2- and neu-transfected NIH/3T3 cells

**Fig. 2:**
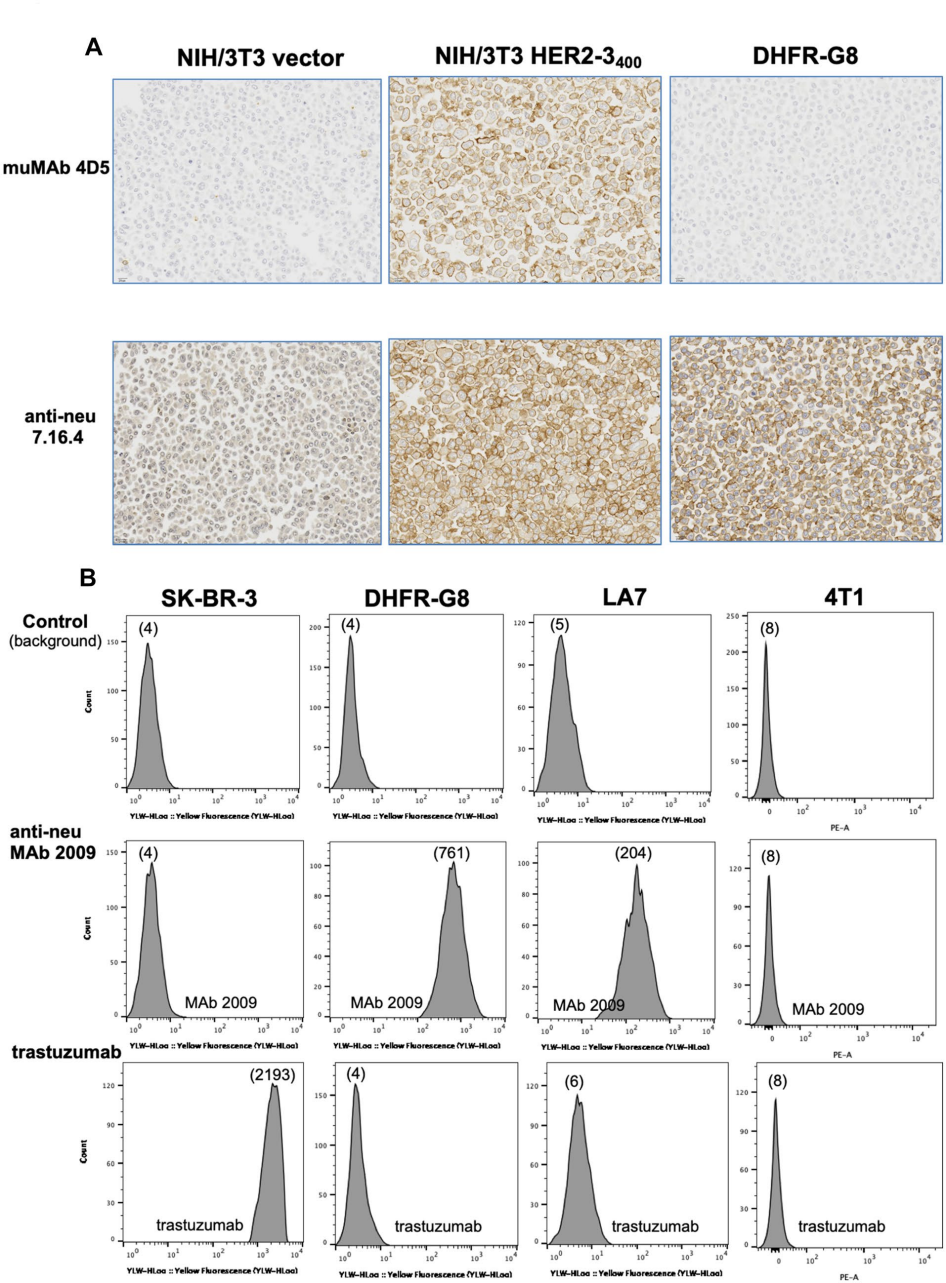
Anti-neu and anti-HER2 antibody binding to HER2-amplified human breast cancer cell line SK-BR-3, HER2-transfected NIH/3T3, neu-expressing DHFR-G8, rat breast tumor cells LA7, and murine breast tumor cells 4T1. **A** Immunohistochemical analysis demonstrates binding of muMAB 4D5 to human HER2 on HER2-transfected NIH/3T3 cells (NIH/3T3 HER2-3_400_), but not to neu on DHFR-G8 cells, upper panels. MAb 7.16.4 cross-reacts with human HER2 (NIH/3T3 HER2-3_400_) and neu (DHFR-G8), as previously reported (Zhang et al.) in lower panels. Low binding to endogenous neu is also observed in NIH/3T3 vector cells with 7.16.4. **B** Trastuzumab binds selectively to human HER2 on SK-BR-3 cells, not to neu on DHFR-G8, LA7, or 4T1 cells, as assessed by FACS analysis. Anti-neu MAb 2009 shows strong binding to neu on DHFR-G8 and LA7 cells, but not to mouse (4T1) or human HER2 (SK-BR-3). Numbers in parentheses are MFI (mean fluorescent intensities)

Immunohistochemistry was performed for independent verification of antibody binding selectivity. Cells of the same genetic background were selected: NIH/3T3 mouse fibroblasts engineered to express either c-neu (DHFR-G8) or human HER2 (NIH/3T3 HER2-3_400_). NIH/3T3 cells with empty vector served as the control. MAb 7.16.4 was utilized for these studies, as this MAb cross-reacts with neu and human HER2 [30]. For consistency of immunohistochemistry reagents, the murine parent of trastuzumab, muMAb 4D5 was used for HER2 detection. In agreement with our FACS analyses, muMAb 4D5 demonstrated robust membrane staining in mouse cells transfected to overexpress human HER2 (NIH/3T3 HER2-3_400_), while muMAb 4D5 did not react with neu in DHFR-G8 cells (Fig. 2A, upper panels). Consistent with previous reports, MAb 7.16.4 detected both human HER2 and rodent neu in NIH/3T3 HER2-3_400_ and DHFR-G8 cells [30], respectively (Fig. 2A, lower panels). Slight staining observed in NIH/3T3 vector cells by MAb 7.16.4 is likely due to expression of endogenous neu in this mouse cell line.

### Binding of trastuzumab versus MAb 2009 on HER2-positive breast cancer cells, neu-overexpressing mouse fibroblasts, and rat or mouse mammary tumor cells by FACS

FACS studies on live, non-permeabilized, unfixed cells were performed to compare cell surface binding of anti-HER2 antibodies to MAb 2009 on cells expressing human HER2 (HER2-amplified human breast carcinoma line SK-BR-3), rat neu (rat fibroblasts DHFR-G8; rat breast tumor cells LA7 and RBA), and mouse neu (4T1 and EMT6 mouse breast tumor cells; HC11 mouse mammary epithelial cells). Cells were incubated with antibodies at 4 °C to prevent antibody-mediated receptor internalization. Trastuzumab demonstrated robust binding to SK-BR-3 cells, showing a 3-log shift in fluorescence intensity compared to background control (Fig. 2B, left panels), with no detectable binding of MAb 2009. Binding of MAb 2009 was demonstrated in DHFR-G8, LA7 and RBA cells (Fig. 2B, Supp. Fig. S1). Binding of trastuzumab to rat (DHFR-G8, LA7, RBA) or murine (4T1, EMT6, HC11) cells was undetectable. Interestingly, MAb 2009 showed no binding to murine neu, suggesting amino acid differences in the epitope on rat versus murine neu required for binding. Pertuzumab also demonstrated selective binding to human HER2 versus neu, similar to trastuzumab (Supp. Fig. S2). Together, these data support binding selectivity of trastuzumab to human HER2 versus rat or mouse neu.

### Radiolabeled trastuzumab and MAb 2009 binding

**Fig. 3:**
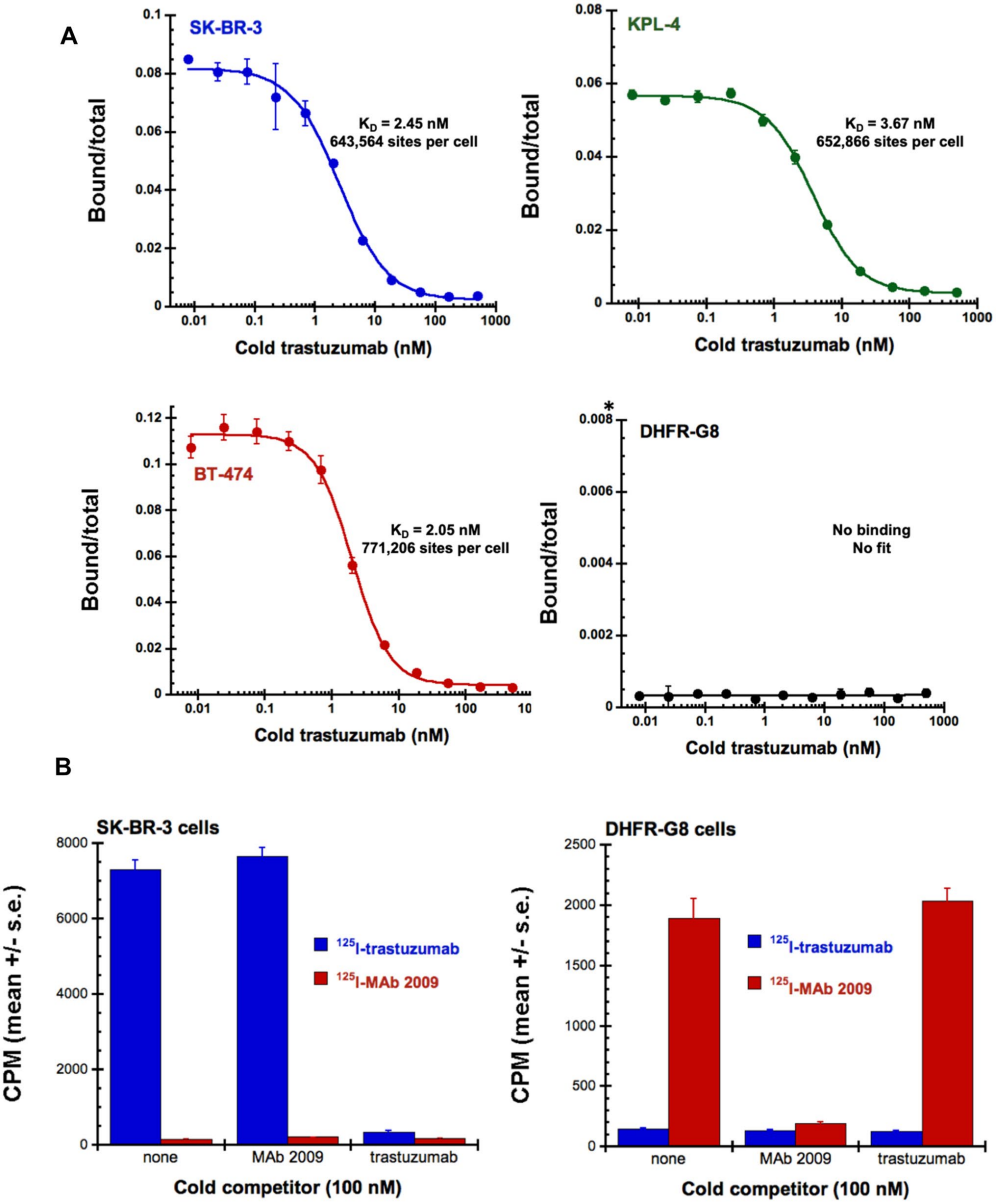
**A** Scatchard analysis of ^125^I-trastuzumab binding to human HER2-amplified breast cancer cell lines SK-BR-3, KPL-4, and BT-474; binding of ^125^I-trastuzumab to neu-overexpressing DHFR-G8 cells was not detectable. Data points represent the mean of 3 replicates ± standard error of the mean (s.e.). **B** Binding specificity of trastuzumab and MAb 2009 to human HER2 on SK-BR-3 cells (left panel) versus neu-expressing DHFR-G8 cells (right panel). Cells were incubated with either ^125^I-MAb 2009 or ^125I^I-trastuzumab (at final concentrations of 45.8 and 43.5 pM, respectively) in the absence or presence of 100 nM unlabeled Mab 2009 or 100 nM unlabeled trastuzumab. Bound counts are plotted as the mean of 4 replicates ± s.e

Radiolabeled ligand binding assays represent one of the most sensitive methods for determining binding parameters (binding sites per cell, binding affinities). Trastuzumab was radiolabeled with ^125^I to high specific activity and competition binding assays were performed on HER2-amplified human breast carcinoma lines SK-BR-3, KPL-4 and BT-474 and DHFR-G8. Scatchard analysis was performed to determine receptor sites per cell and equilibrium dissociation constants (K_D_). The K_D_ for trastuzumab was similar on the 3 breast cancer cells (2–3.7 nM), consistent with previous findings [31], as were the number of binding sites per cell (between 644,000 and 771, 200). In contrast, binding of ^125^I-trastuzumab was not detectable on DHFR-G8 cells (Fig. 3A). Additional competition binding studies were performed to investigate whether trastuzumab and MAb 2009 compete with each other for binding human HER2 or neu. SK-BR-3 and DHFR-G8 cells were incubated with ^125^I-trastuzumab or ^125^I-MAb 2009, in the absence or presence of excess unlabeled competitor antibody. Binding of ^125^I-trastuzumab to SK-BR-3 cells was effectively competed with unlabeled trastuzumab, as expected, while ^125^I-MAb 2009 did not bind SK-BR-3 cells and did not compete with trastuzumab binding (Fig. 3B, left panel). Consistent with FACS and IHC studies, there was no specific binding of ^125^I-trastuzumab to neu-expressing DHFR-G8 cells. Specific binding of ^125^I-MAb 2009 to DHFR-G8 cells was observed, and could be competed with unlabeled MAb 2009, but not unlabeled trastuzumab (Fig. 3B, right panel). Taken in full, results of radiolabeled antibody competition binding studies strongly support that trastuzumab does not bind the neu receptor.

### Structural and mutational analysis of the trastuzumab binding interface on HER2 ECD domain IV

**Fig. 4:**
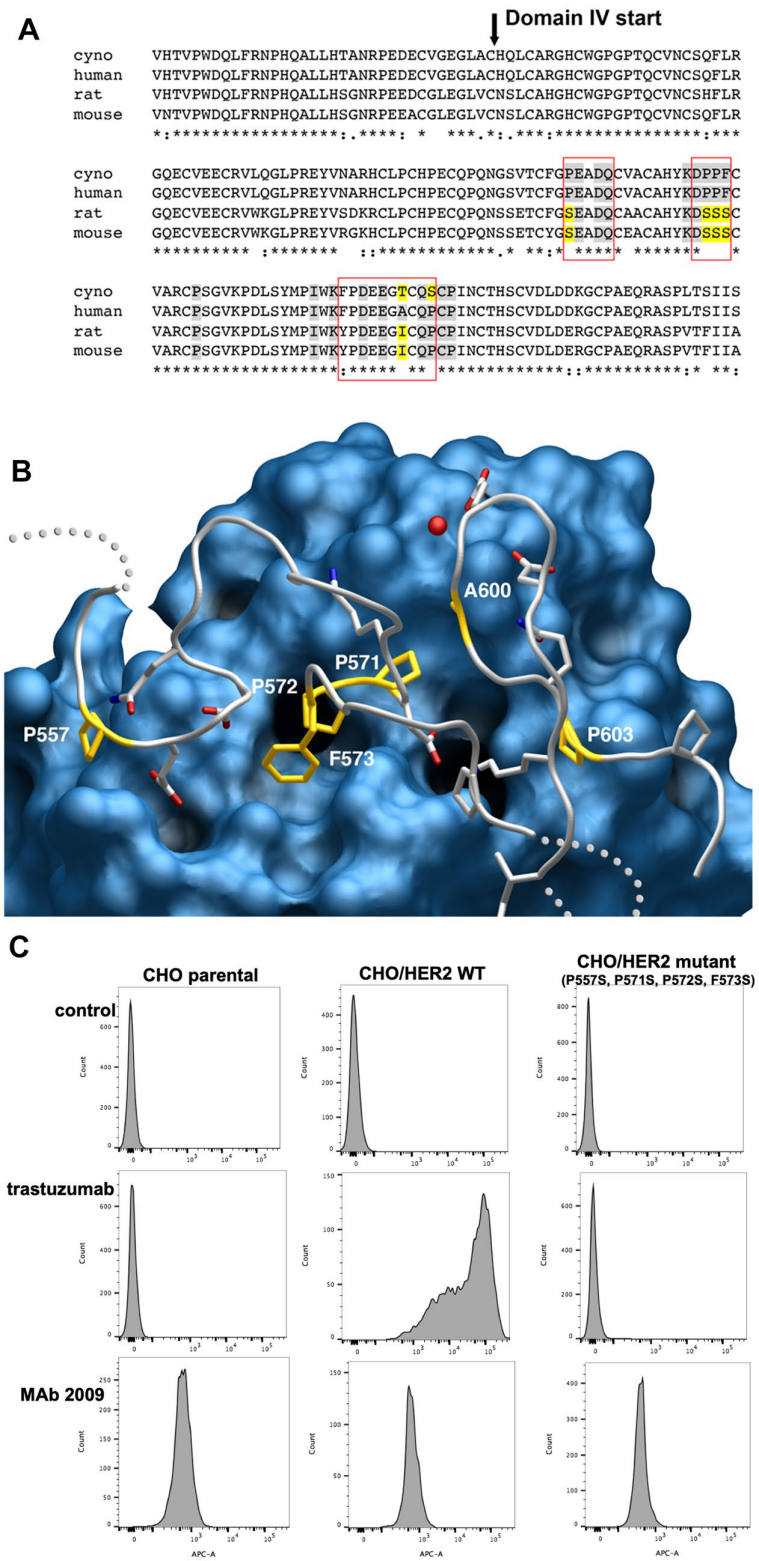
Amino acid sequence alignment (**A** top) for cynomolgus monkey (EHH58073.1), human (NP_004439.2), rat (NP_058699.2), and mouse (NP_001003817.1) HER2/*neu* of the region recognized by trastuzumab. Shaded regions contact the antibody, with gray coloring representing conservation and yellow representing differences with respect to the human sequence. **B** crystal structure (1N8Z) of the human HER2: trastuzumab interface [32]. HER2 is colored gray, except at interface residues differing from the human sequence (gold). The trastuzumab Fab is depicted as a blue surface. **C** Mutation of prolines 557, 571, 572, and phenylalanine 573 from human HER2 to serine residues present in *neu* abolish binding of trastuzumab versus wildtype HER2 in transfected CHO cells

The co-crystal structure between trastuzumab Fab and HER2 revealed the contact residues (i.e., epitope) on HER2 [32]. Amino acid sequence alignment for human, cynomolgus monkey, rat and mouse HER2/neu revealed that the trastuzumab binding epitope is not conserved in rodent species. It was previously shown that mutations in the pertuzumab epitope that substituted the two important sidechain contact residues in human HER2, Leu 295 and His 296, with the neu residues-L295P, H296N or L295P/H296N—abolished pertuzumab binding [14]. As this type of analysis was not performed for trastuzumab, we next investigated the impact of sequence differences on trastuzumab binding to human HER2 versus neu. Sequence homology in domain IV between human HER2 and rodent neu is 84.4% (22/141 amino acid differences). The crystal structure of the trastuzumab Fab fragment bound to domain IV of the HER2 ECD [32] demonstrated 19 amino acid sidechains of domain IV that form the epitope recognized by the trastuzumab fab (Fig. 4A). Of these residues, 5 differ in rodent neu, including a central patch of 3 continuous residues, Pro-Pro-Phe (PPF 571-573) in human HER2 versus serines (SSS) in neu. An additional proline residue in human HER2, amino acid 557, is also replaced by serine in rodent neu (Fig. 4A, B). To investigate impact of these amino acid substitutions on trastuzumab binding, we engineered CHO cells to express high levels of either wildtype HER2 or a quadruple mutant P557S/P571S/P572S/F573S, to reflect the differences in the trastuzumab binding interface between human HER2 and neu. As expected, trastuzumab exhibited robust binding in CHO cells expressing high levels of wildtype human HER2, with no detectable binding to CHO parental cells. Mutation of prolines 557, 571 and 572, and phenylalanine 573 from human HER2 to serine residues present in neu completely abolish trastuzumab binding (Fig. 4C). The presence of the serine patch in neu shortens the sidechains and inserts polarity into a hydrophobic region of the fab surface, likely preventing interaction between neu and trastuzumab. Moreover, conformational flexibility of the neu serine sequence in this region is likely increased, as compared to conformational rigidity imposed by three prolines and one phenylalanine. The presence of the four mutations from rat/mouse neu did not confer binding activity of MAb 2009, possibly indicating a different binding epitope for this antibody.

Domain IV sequence homology for human HER2 versus cynomolgus monkey is 98.6% (2/141 amino acid differences). The primate sequence differs from human at only 2 of the 19 epitope positions. Both sites form less substantial contact surfaces with the fab and occur at the periphery of the interaction surface. The two amino acid differences between human and cynomolgus monkey HER2 domain IV do not affect binding of trastuzumab IgG1 [33, 34].

### Immunoblot studies for interaction of trastuzumab and MAb 2009 with HER2 and neu

**Fig. 5:**
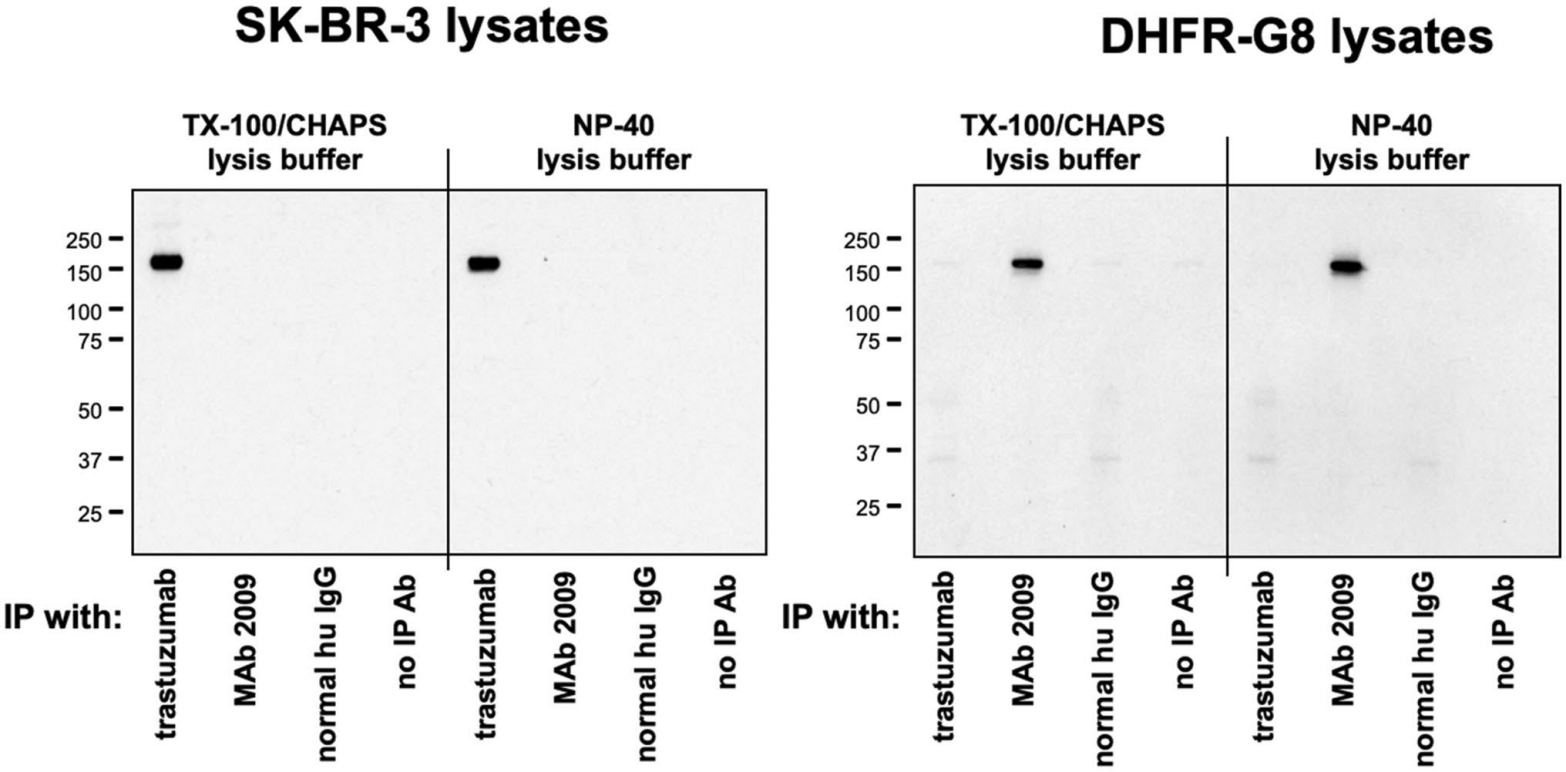
IP/Western blot analyses demonstrate that trastuzumab interacts only with human HER2 on SK-BR-3 cells (left panel), while MAb 2009 detects neu on DHFR-G8 cells (right panel) using our standard blotting conditions (TX-100/CHAPS) and those described by Lorenzo et al. (26) (NP-40). IP antibodies are listed for each lane; membranes were blotted with antibody D8F12 XP, which recognizes both human HER2 and neu

Our preferred methods for assessing antibody-receptor interactions are FACS and radioligand binding assays. A significant advantage of these methods is the use of live, intact cells to assess cell surface binding (the relevant interaction for therapeutic antibodies), with no need for detergent solubilization or other chemical/mechanical manipulation of cells. However, because numerous publications cite, but do not independently verify, publications that assessed binding of trastuzumab to neu or HER2 by immunoblot, we performed similar studies. Our IP/immunoblot method was compared side by side with cited methods [15], with the exception that the same antibody concentration was used for all IP conditions (2 μg) different from published methods IP antibody concentrations of 5 μg for anti-neu [Santa Cruz antibody not specified] versus 10 μg for trastuzumab or MAb Erb-hc-Ab [15]; 0.2 μg anti-neu 7.16.4 versus 40 μg anti-HER2 4D5 [30]. IP antibody concentrations were not specified in related publications [25, 26]. An additional difference in methods was the use of a highly dense cell volume (15 million cells/mL) and non-ionic/non-denaturing detergent only (0.5% NP-40) for cell lysis [15, 26] compared to our procedure of lysing 3 million cells/mL, in 1% Triton X-100 (non-ionic/non-denaturing) plus 1% CHAPS (a zwitterionic detergent that is stronger than non-ionic detergents, therefore better for solubilization of cells and disruption of protein–protein interactions [35]. Consistent with our FACS data, our immunoblot studies, under both conditions, demonstrate that trastuzumab does not immune-precipitate neu in DHFR-G8 cells (Fig. 5, right panel). Immune precipitation of neu was demonstrated only with MAb 2009. As expected trastuzumab, but not MAb 2009, immune-precipitated HER2 in SK-BR-3 cells (Fig. 5, left panel). Thus, we were not able to reproduce previous reports that trastuzumab immune-precipitates rodent neu [15, 25]. Non-specific bands observed with immunoblot analysis can arise from a number of experimental conditions, including excessive antibody concentrations or incomplete dissolution of cells and protein complexes [36]. These conditions may have contributed to the appearance of bands incorrectly interpreted as resulting from interaction of trastuzumab with rodent neu in previous reports.

### In vitro cell viability, uptake and catabolism studies in human hepatocytes

**Fig. 6:**
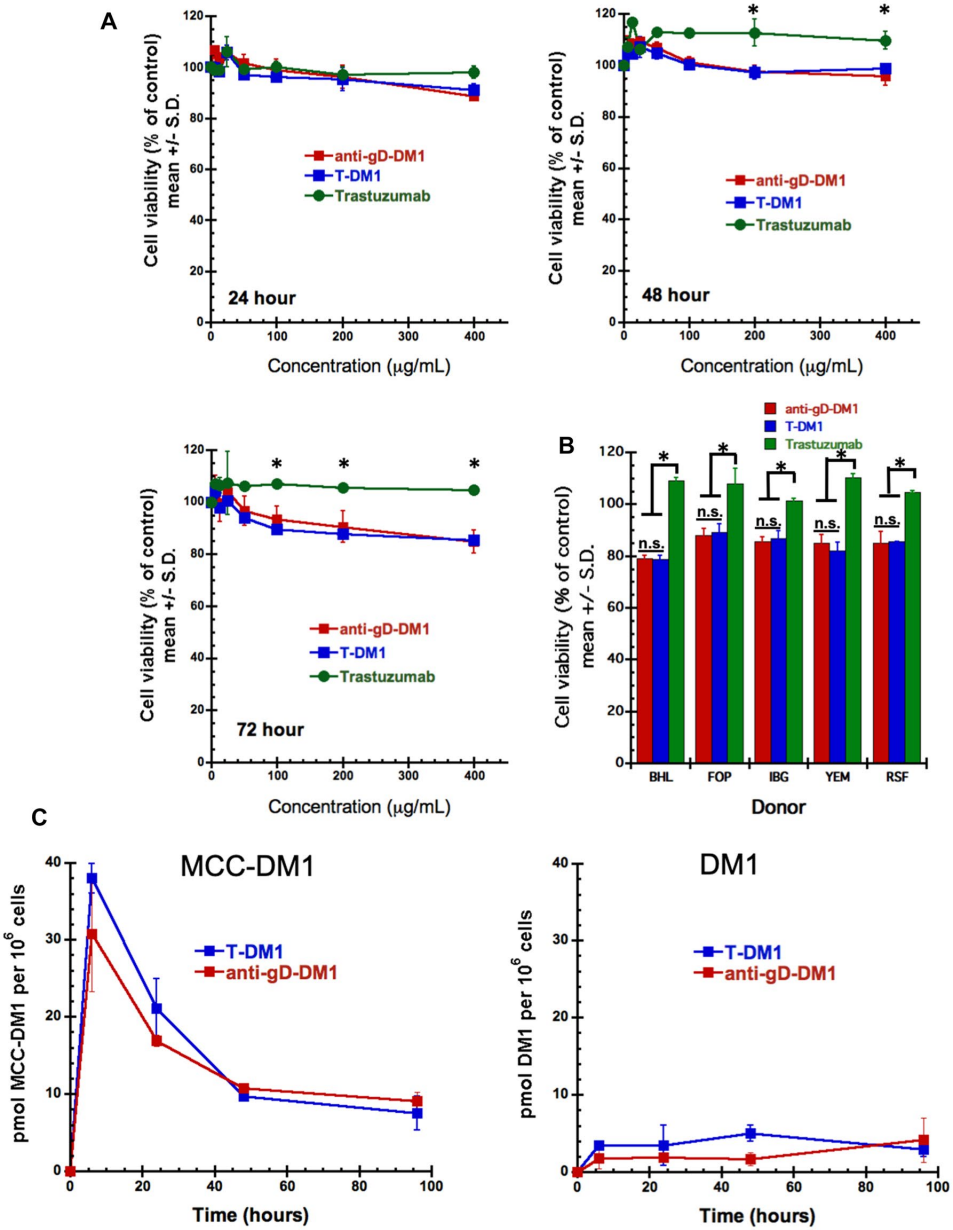

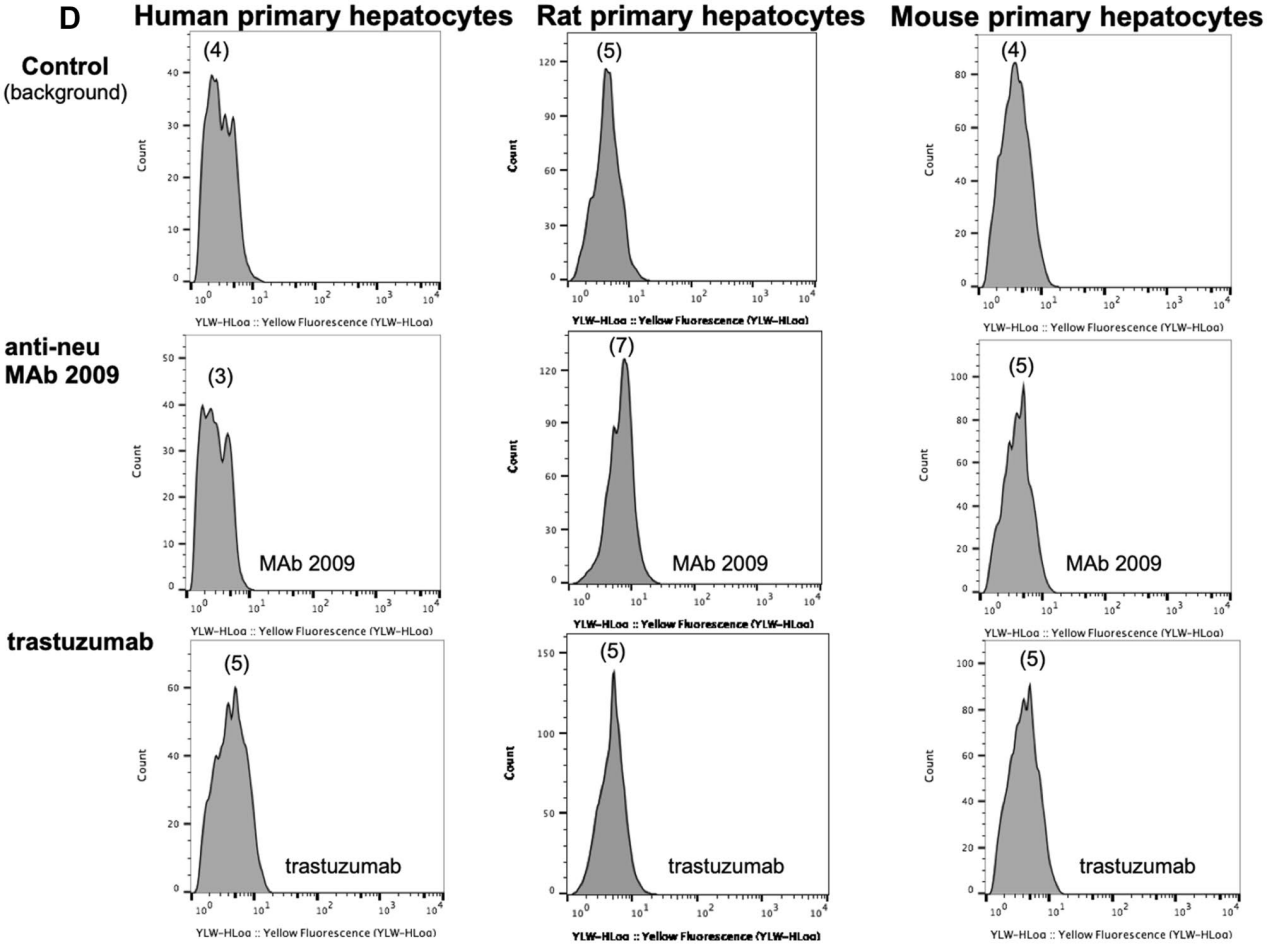
Cytotoxicity assessment of trastuzumab, T-DM1, and anti-gD-DM1 (control ADC) and measurement of ADC catabolites in human hepatocytes. **A** Primary human hepatocytes (lot RSF) were treated for 24, 48, and 72 h with varying concentrations of trastuzumab, T-DM1, or anti-gD-DM1. Cell viability was assessed by measuring total cellular ATP content and reported relative to vehicle controls. Data represent means ± standard deviation (S.D.), with *n* = 3 per condition. Statistical differences (*p < 0.05) were observed between trastuzumab and either T-DM1 or anti-gD-DM1 at individual concentrations. **B** Cytotoxicity of trastuzumab, T-DM1, or anti-gD-DM1 in five lots (lots BHL, FOP, IBG, YEM, and RSF) of plated primary human hepatocytes following treatment with 400μg/mL for 72 h. Statistical differences were observed between the means of T-DM1 or anti-gD-DM1 versus the mean of trastuzumab across all lots of hepatocytes (*represents *p* < 0.05.; n.s. = not significant). **C** LC–MS/MS measurement of catabolites formed in human hepatocytes treated with T-DM1 or anti-gD-DM1 over 4 days. **D** FACS analysis demonstrates no trastuzumab or MAb 2009 binding to human, rat, or mouse hepatocytes. Numbers in parentheses are MFI

Biological studies were performed to assess T-DM1 activity in human hepatocytes, in particular to address our concerns regarding the conclusions of Yan et al. [25] that effects of T-DM1 in mouse models of hepatotoxicity are HER2-mediated via trastuzumab binding. Primary human hepatocytes were obtained from 5 different donors and treated with T-DM1, anti-gD-DM1 (non-targeted ADC), or trastuzumab. Concentrations ranged from 6.25 to 400 μg/mL, with the highest concentration 40-fold higher than typically used in tumor cell viability assays [37]. While trastuzumab had no effect on hepatocyte viability (Fig. 6A), treatment with both T-DM1 and anti-gD-DM1 resulted in a 15% reduction in viability at concentrations of 200 and 400 μg/mL. Of note, these concentrations are far in excess of breast cancer patient T-DM1 serum concentrations, where C_max_ values are typically under 100 μg/mL [38]. Moreover, there was no difference between T-DM1 and anti-gD-DM1, indicating that the modest reduction in cell viability was not HER2-mediated but DM1-moiety related. Figure 6B shows similar results across 5 different hepatocyte lots.

Because the liver is a primary organ for drug metabolism, we performed additional studies on human hepatocytes to investigate uptake and catabolism of T-DM1 and anti-gD-DM1 by measuring the catabolites DM1, MCC-DM1 and Lys-MCC-DM1, by LC-MS/MS, produced after treatment. As shown in Fig. 6C, similar profiles and levels of catabolites were detected in hepatocytes exposed to both T-DM1 and anti-gD-DM1. MCC-DM1 appeared to be the main catabolite (left panel), with much lower levels of DM1 detected (right panel). Peak MCC-DM1 levels were demonstrated after 6 h of treatment with both T-DM1 and anti-gD-DM1, with decreasing levels over the remainder of the study. These data are similar to those reported for human HER2-positive breast cancer patients, where MCC-DM1 and DM1 were the primary catabolites measured in plasma [27]. In summary, as catabolite levels were not different between anti-HER2- and anti-gD DM1-containing ADCs, these data provide additional evidence for non-HER2-mediated uptake of ADCs in human hepatocytes. To support these observations, FACS studies were performed on human, rat and mouse hepatocytes. No binding was observed for trastuzumab or MAb 2009 on primary human, rat or mouse hepatocytes (Fig. 6D), or for pertuzumab (Supp. Fig. S3). These findings are consistent with very low HER2 expression levels in normal human liver compared to other normal tissue (Genotype-Tissue Expression Portal, https://www.gtexportal.org; Supp. Fig. S4; and the Human Protein Atlas, https://www.proteinatlas.org/ENSg00000141736-ERBB2/tissue).

## Discussion

Elucidating mechanisms of action and toxicity are key to development of therapeutics. For antibody-based therapeutics, selection of relevant binding species is integral for understanding targeted versus non-targeted safety signals. Inclusion of appropriate non-targeted controls for studies with antibody–drug conjugates in particular is essential to determine toxicities mediated by antigen binding of the ADC versus non-antigen-mediated uptake, which occurs in numerous tissues and organs, and can lead to signals mediated by the cytotoxic drug component of the ADC. Because the anti-HER2 antibody trastuzumab, as well as trastuzumab-containing ADCs such as T-DM1 are approved therapeutics, numerous studies have been performed to understand safety events associated with administration, i.e., cardiac toxicity with trastuzumab; thrombocytopenia and hepatoxicity with T-DM1. Given limited data whether trastuzumab binds rodent neu, investigators over the years attempted to study cardiac effects of trastuzumab in mouse models [15, 16, 18, 19, 39, 40], as well as effects of T-DM1 in mice to model hepatoxicity [25], assuming trastuzumab/T-DM1 would mediate target-specific toxicity in rodents. Results from these studies are difficult to interpret due to lack of appropriate non-targeted control antibodies or ADCs, and, more so, given our data demonstrating that trastuzumab does not bind rodent neu. The few publications that describe trastuzumab binding to neu use immunoprecipitaton studies with no IgG control [15, 17, 18] or that show IP of HER2 even with the IgG negative control in mouse hepatocytes [25]. Results of binding studies of trastuzumab and T-DM1 on human and mouse hepatocytes by immunofluorescence microscopy [25] are equally difficult to interpret as the fluorescent signal shown was not circumferential/cell surface as would be expected for HER2 staining, but was localized in one or two cellular protrusions, and appeared intracellular. Moreover, the use of high concentrations that result in non-target-mediated uptake (50 and 100 μg/mL) likely lead to the intracellular T-DM1 signal reported. Our work includes detailed studies of antibody binding to cell surface HER2/neu in live, intact cells by flow cytometry and Scatchard analysis, as these methods are unencumbered by conditions which can induce potential artifacts (lysis buffers and other conditions used for immunoblot; fixation reagents for microscopy). Additionally, we undertook a series of experiments to investigate target-dependent cytotoxicity, uptake and catabolism of T-DM1 by comparing with a non-targeted DM1 ADC in human hepatocytes.

We first generated a panel of anti-neu monoclonal antibodies for comparison to 4D5/trastuzumab. The majority of clones demonstrated robust binding to neu, with no binding to human HER2, HER3 or HER4. Binding of clone 2009 to neu was comparable or better than the benchmark literature antibody 7.16.4. Binding of 7.16.4 and muMAb 4D5 on neu- and human HER2 overexpressing cells by immunohistochemistry showed selectivity of 4D5 binding for human HER2 on 3T3/HER2 cells versus no binding to DHFR-G8. Flow cytometry studies confirmed selectivity of humanized 4D5, trastuzumab, for human HER2 (SK-BR-3 cells) versus rat or mouse neu. Interestingly, MAb 2009 showed preferential binding to rat neu compared to mouse, indicating that, despite high homology in the ECD (95%) for rat and mouse neu, sequence differences are sufficient to impact binding. In addition to flow cytometry and IHC, binding studies were performed with radiolabeled antibodies as this method is considered one of the most sensitive and specific types of binding assays. Scatchard analysis with ^125^I-trastuzumab confirmed specific binding of trastuzumab to HER2-positive breast cancer cells, with no detectable binding to neu-expressing cells. Competition binding assays demonstrated that trastuzumab, but not MAb 2009, competed with ^125^I-trastuzumab binding on SK-BR-3 breast cancer cells, while MAb 2009, but not trastuzumab, competed with ^125^I-MAb 2009 binding on neu-expressing DHFR-G8 cells.

Structural studies by Franklin et al. elucidated binding of 2C4/pertuzumab to HER2 ECD sub-domain II. Substitution of the two amino acid residues in the HER2/pertuzumab binding interface with the corresponding neu residues abolished pertuzumab binding to HER2 [14]. We took a similar approach for trastuzumab by replacing 4 amino acids in the trastuzumab binding interface with the corresponding residues in rat and mouse neu. Similar to the findings with pertuzumab, trastuzumab binding to HER2 was abolished by substitution of the neu amino acids into the binding interface.

Finally, we sought to determine if trastuzumab binding to neu could be detected by immunoblot, as previously reported [15, 18, 25]. Under experimental conditions similar to those reported, as well as using our own protocol, we could not detect binding of trastuzumab to neu. In total, we have demonstrated by flow cytometry, immunohistochemistry, competition assays using radiolabeled antibodies, and structure-based studies that trastuzumab does not bind rodent neu.

To address the report that T-DM1 mediates hepatotoxicity in mice via HER2-mediated mechanisms, we investigated anti-proliferative activity, uptake and catabolism of T-DM1 in human hepatocytes, as well as binding in human, rat and mouse hepatocytes. No detectable binding of T-DM1 was observed in hepatocytes from the 3 different species, consistent with our data showing no binding of trastuzumab to rodent neu, with IHC data demonstrating HER2 expression in biliary epithelial cells but not hepatocytes [41], as well as with data from publicly available genomic and proteomic databases that HER2 levels are very low in normal human liver. This raises a question regarding interpretation of data from Yan et al. [25] on hepatotoxic effects of T-DM1 in mice. In properly controlled experiments, we demonstrated that a non-targeted antibody-DM1 conjugate showed the same activity in hepatocytes as T-DM1. Both conjugates modestly reduced hepatocyte viability, albeit at concentrations 2–4 fold above pharmacologically relevant levels [38]. Moreover, uptake and catabolite production were comparable between both targeted (HER2) and non-targeted (gD) DM1 conjugates. These data indicate that the pharmacologic effects were mediated by DM1 in a target-independent manner. As the Yan paper lacks a non-targeted DM1 ADC control, the data are difficult to interpret. The cell culture studies utilized high T-DM1 concentrations (5 and 10 μg/mL) known to elicit non-targeted activity in breast cancer cells and primary cells that are HER2 negative [37, 42]. It is important to note that normal epithelial cells or carcinoma cells that are IHC 0/HER2 negative can express low levels of HER2. Moreover, in vivo hepatic effects of T-DM1 in mice were observed at the high dose of 30 mg/kg (> 8 times the human clinical dose). Without a control DM1 ADC, it is impossible to interpret these effects as ‘HER2-mediated’. A more recent paper from these authors then stated opposing conclusions, that the effects were HER2 independent and mediated by DM1 [43], and that DM1 in fact binds to a cell surface cytoskeletal protein to mediate hepatocyte toxicity.

A number of trastuzumab-based ADCs have entered the clinic since the approval of T-DM1. Non-human primates are consistently utilized for safety studies as the relevant binding species, due to lack of binding of trastuzumab to rodent neu [33, 34, 44, 45]. Detailed toxicologic evaluation in cynomolgus monkey (as a binding species) and rats (as a non-binding species) demonstrated concordant safety findings in animals treated with T-DM1 or free DM1, leading to the conclusion that toxicities were target-independent and reflected the mechanism of action of DM1 [33]. Investigation into potential mechanisms of T-DM1-induced thrombocytopenia revealed effects on megakaryocytes mediated via either FcγRIIa uptake [22] or macropinocytosis [23], as neither megakaryocytes or platelets express HER2. Fc receptors mediate antibody/ADC uptake into other tissues as well [46]. In fact, it is widely accepted that the majority of ADC toxicities are not target dependent, but are payload associated due to non-target-mediated uptake of ADCs into normal tissue. Thus, ADCs targeting different tumor antigens, but with the same payload, generally elicit similar toxicities [47–50]. For example, the frequency of hepatotoxicity across different ADCs can be explained by the extensive clearance and catabolism of ADCs via the liver reticuloendothelial system [46, 48].

In summary, through detailed binding and structural studies, as well as pharmacologic studies in hepatocytes, we have provided strong evidence that trastuzumab does not bind rodent neu, and that effects of T-DM1 in liver cells are not HER2-mediated. Development of HER2-directed antibody therapies, comprised of trastuzumab or other HER2 antibodies, is an active area of drug research. The data herein may guide the design of safety and efficacy studies with trastuzumab-based therapies as well as potentially reduce animal use for unnecessary experimentation.

## Supplementary Information

Below is the link to the electronic supplementary material.

Supplementary file1 (DOCX 6238 KB)

## Data Availability

Upon request of corresponding author.
