# Supplementary material for: Trastuzumab does not bind rat or mouse ErbB2/neu: implications for selection of non-clinical safety models for trastuzumab-based therapeutics
Source: Breast Cancer Res Treat. 2021 Oct 27;191(2):303–17. doi: 10.1007/s10549-021-06427-w (PMC8763818; doi:10.1007/s10549-021-06427-w)
Supplement: Supplementary file 1 — Supplementary file1 (DOCX 6238 KB) [file 10549_2021_6427_MOESM1_ESM.docx]

**Supplemental methods**

**Cell lines and reagents**: SK-BR-3 and BT-474 human breast carcinoma cell lines, DHFR-G8, CHO-K1 (Chinese Hampster Ovary, clone K-1), RBA and LA7 rat mammary carcinoma cell lines, HC11 normal mouse epithelial cells, and 4T1 and EMT6 mouse mammary carcinoma cell lines were obtained from the American Type Culture Collection and used within 4-8 passages. DHFR-G8 are NIH/3T3 mouse fibroblasts co-transfected with rat neu DNA and the dihydrofolate reductase (DHFR) gene to achieve neu amplification in the presence of methotrexate [1]. KPL-4 human breast carcinoma cells were a gift from J. Kurebayashi[2]. KPL-4 cells were authenticated using short tandem repeat loci analysis at Genetica DNA Laboratories using the reference KPL-4 profile. Cells were maintained as previously described[3]. NIH/3T3 vector and NIH/3T3 HER2-3_400_ were made at Genentech[4]. Methotrexate (300 nM) was included in the medium for DHFR-G8 cells to maintain high neu expression. Geneticin (400 μg/mL) was added to culture medium for the NIH/3T3 vector and HER2-transfectants. Cryopreserved primary human hepatocytes, InVitroGro HT™ thawing medium, InVitroGro CP™ plating medium and 1% Torpedo™ Antibiotic Mix were obtained from BioreclamationIVT. For FACS assays, human and rat hepatocytes were from ThermoFisher and mouse hepatocytes from GIBCO, and were cultivated per vendor instructions. Trastuzumab, pertuzumab, trastuzumab emtansine, anti-HER2 MAb 4D5 (muMAb 4D5; ATCC CRL-10463), anti-HER3 MAb 1511, anti-HER4 MAb 1440 (ATCC HER4.1C6.A11, PTA-2829), anti-*neu* MAbs, anti-gD (herpes simplex virus glycoprotein D) MAb 1766, and anti-gD 1766-MCC-DM1 were produced at Genentech. Anti-neu MAb 7.16.4 was obtained from Calbiochem. Anti-mouse and anti-human IgG-FITC, and normal human IgG were from Jackson ImmunoResearch. Anti-HER2/ErbB2 D8F12 XP was obtained from Cell Signaling Technology. For immunohistochemistry (IHC), Vectastain Elite ABC Kit (Vector Laboratories) was utilized. Other reagents include NBF (10% neutral buffered formalin, Sigma-Aldrich), CellTiter-Glo Assay™ (Promega), Cell Dissociation Non-Enzymatic Solution (Sigma-Aldrich), Lipofectamine 3000 (Invitrogen), Cell Lysis Buffer (Cell Signaling Technologies), complete EDTA-free protease inhibitor cocktail (Roche), and Protein-A/G Ultralink resin (ThermoFisher Scientific). Human HER2 wildtype and mutant expression constructs were from GenScript USA.

**Fluorescence-activated cell sorting (FACS) analysis**

Cells were detached with Cell Dissociation Solution, centrifuged and resuspended in assay buffer (2% fetal bovine serum [FBS] in phosphate-buffered saline [PBS]) at a density of 1 million cells/mL. Cells were aliquoted into 0.1 mL volumes and incubated with 10 μg/mL anti-HER2 or anti-neu MAbs for 1 hour at 4° C. Cells were then centrifuged, cell pellets washed, and FITC (fluorescein isothiocynate)-labeled secondary antibodies (anti-mouse or anti-human IgG) added for 1 hour at 4° C. These assays were performed on live, non-permeabilized, unfixed cells. Treatment with antibodies at 4° C prevents antibody-mediated receptor internalization. Cells were centrifuged, washed, resuspended in assay buffer and fluorescence assessed using a Guava EasyCyte Flow Cytometer (EMDMillipore). Histograms were plotted using FlowJo FACS data analysis software (FlowJo, LLC). For CHO-HER2 cells, samples were analyzed using an LSRFortessa cell analyzer (BD Biosciences).

**Immunohistochemistry**

NIH/3T3 cells expressing rodent c-neu (DHFR-G8), human HER2 (NIH/3T3 HER2-3/_400_) or the vector control NIH/3T3 were utilized for immunohistochemistry (IHC). Cells were detached non-enzymatically, washed 3 times with PBS, fixed with NBF for embedding into paraffin, and sectioned. Staining was carried out using Vectastain Elite ABC kit. Briefly, sections were deparaffinized and hydrated, blocked with KPL Blocking Solution and digested at 37º C using 0.4% pepsin in 0.1N NaOH for 10 min, 1 mg/mL trypsin in CaCl_2_ plus 0.1N NaOH for 15 min, then at room temperature with 0.05% saponin in water for 30 min. After rinsing with PBS and blocking endogenous IgG binding sites with 10% normal serum, sections were incubated with 10 μg/mL muMAb 4D5 or 7.16.4 overnight at 4ºC. Reaction with biotinylated secondary antibody, ABC Reagent and peroxidase substrate were performed according to manufacturer’s instructions. Sections were counterstained with Mayer’s hematoxylin, rinsed, dehydrated, and mounted.

**Binding studies with radiolabeled antibodies**

Scatchard analysis was performed at Invicro Centre for Molecular Oncology, London, UK. Trastuzumab was labeled with ^125^I using the standard Iodogen method [5]. Final radiochemical purity was >99%, with a final concentration of 163.9 pM and specific activity of 17.6 μCi/μg. For homologous competition binding assays, purified radiolabeled antibody was diluted with DMEM (Dulbecco’s Modified Eagle Medium) to yield 200,000 cpm in 25 μL. Unlabeled antibody was diluted to 5000 nM, then serially diluted in binding buffer (RPMI-1640 with 2% FBS, 50 mM HEPES [hydroxyethyl-piperazineethanesulfonic acid], 0.1 % azide, pH 7.2). After blocking 96-well plates with 10% FBS/RPMI for 1 hr at room temperature, the blocking solution was removed, and 25 μL of each cold antibody dilution, 25 μL of radiolabeled antibody, and 25 μL of binding buffer were added to appropriate wells. After detachment with Accutase, cells were centrifuged and re-suspended in binding buffer. Cell suspensions were added to appropriate wells (50,000 cells per well) to give final cold antibody concentrations of 500 to 0.025 nM. Plates were incubated for 2 hr at room temperature. After transferring the contents of the 96-well plate to pre-washed multiscreen filter plates, supernatants were removed and plates washed 4 times with cold binding buffer. Filters were removed and transferred to tubes for gamma counting. Data analysis was performed with GraphPad Prism 5, using the Homologous Competition Binding fit method for non-linear curve fitting to yield binding affinity (K_D_), receptor density (B_max_) and a measure of the fit quality (R^2^).

Binding specificity of trastuzumab and MAb 2009 to human HER2 and rodent neu was determined using SK-BR-3 and DHFR-G8. Cells were seeded at 100,000 per well in 24 well plates and allowed to attach overnight. Medium was removed and replaced with ice cold RPMI containing 2.0 mg/mL BSA, and 232,000 cpm of ^125^I-MAb 2009 (45.8 pM) or 234,000 cpm of ^125I^I-trastuzumab (43.5 pM), either in the absence or presence of 100 nM unlabeled MAbs. Antibodies were radiolabeled by PerkinElmer to initial specific activities of 26.9 μCi/μg (MAb 2009) and 28.6 μCi/μg (trastuzumab). Cells were incubated with antibodies for 3 hr on ice. Unbound counts were removed by washing cells three times with cold RPMI. Cells were then solubilized with 0.1 N NaOH containing 2% SDS and cell-associated counts measured using an LB2111 Multi Crystal Gamma Counter (Berthold Technologies). Each group consisted of 4 replicates.

1. Hung M-C, Schechter A, Chevray P-Y, Stern D, Weinberg R: **Molecular cloning of the neu gene: absence of gross structural alteration in oncogenic alleles**. *Proc Natl Acad Sci USA* 1986, **83**:261-264.

2. Kurebayashi J, Otsuki T, Tang C, Kurosumi M, Yamamato S, Tanaka K, Mochizuki M, Nakamura H, Sonoo H: **Isolation and characterization of a new human breast cancer cell line, KPL-4, expressing the ErbB family receptors and interleukin-6.** *British J Cancer* 1999, **79**:707-717.

3. Lewis Phillips G, Li G, Dugger D, Crocker L, Parsons K, Mai E, Blattler W, Lambert J, Chari R, Lutz R *et al*: **Targeting HER2-positive breast cancer with trastuzumab-DM1, an antibody-cytotoxic drug conjugate**. *Cancer Res* 2008, **68**:9280-9290.

4. Hudziak R, Schlessinger J, Ullrich A: **Increased expression of the putative growth factor receptor p185HER2 causes transformation and tumorigenesis of NIH 3T3 cells**. *Proc Natl Acad Sci USA* 1987, **84**:7159-7163.

5. Unak T, Akgun Z, Yildirim Y, Duman Y, Erenel G: **Self-radioiodination of iodogen**. *Applied Radiation Isotopes* 2001, **54**:749-752.

**Supplemental figures**

**Supplemental Figure 1.** Trastuzumab does not bind rat (RBA) or mouse (EMT6) breast carcinoma cells, or normal mouse epithelial cells (HC11). MAb 2009 binds rat neu (RBA cells).

**
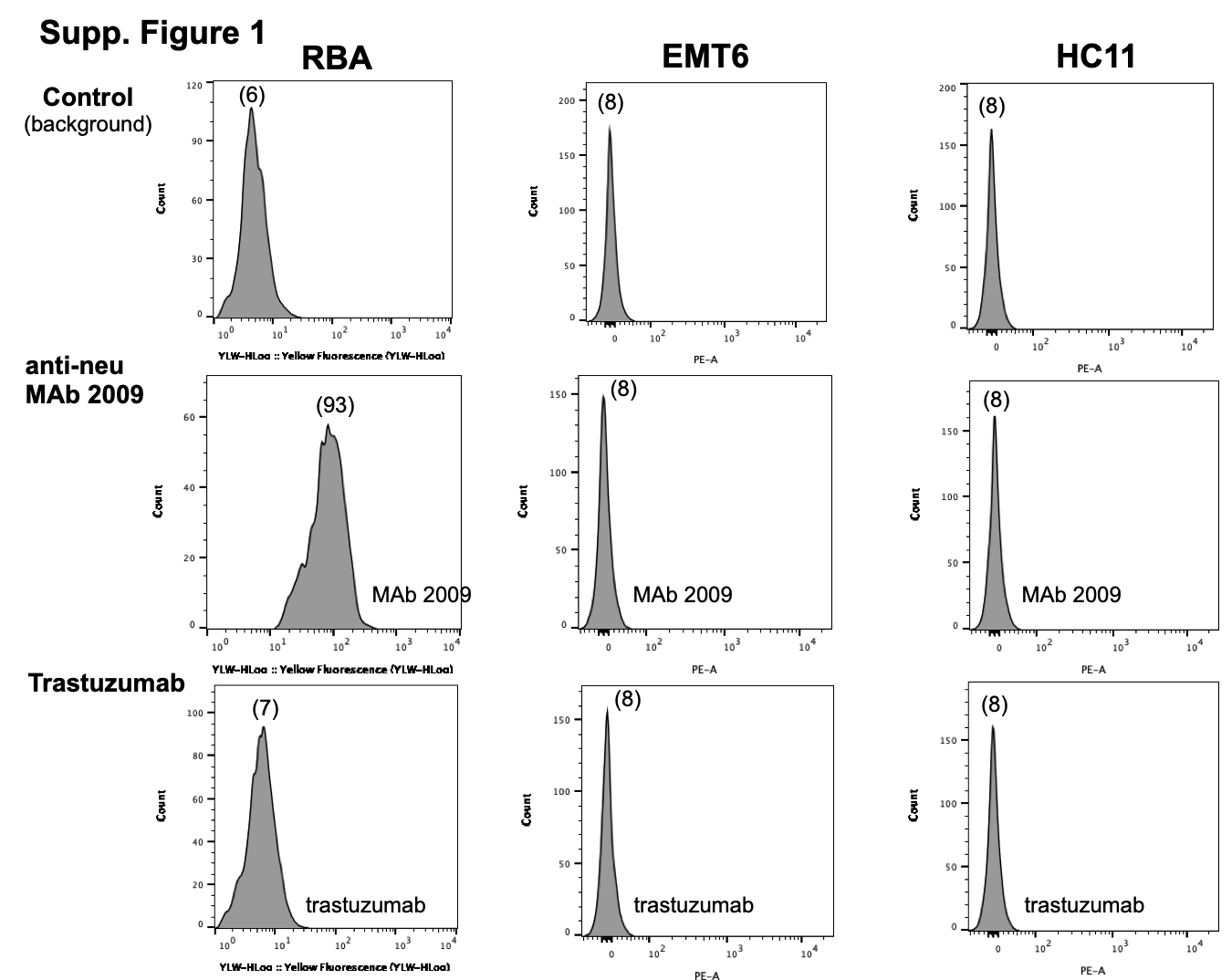
**

**Supplemental Figure 2.** Pertuzumab demonstrates similar binding to human HER2 on SK-BR-3 human breast cancer cells as trastuzumab, but does not bind neu on DHFR-G8 neu-expressing mouse fibroblasts.

**Supplemental Figure 3.** No detectable binding of pertuzumab is observed on human or mouse primary hepatocytes.

**Supplemental Figure 4.** Normal tissue expression of HER2, obtained from [gtexportal.org](file:///Users/nod/Library/Containers/com.microsoft.Word/Data/Downloads/gtex), showing very low expression in liver.
